# A Novel Joint Brain Network Analysis Using Longitudinal Alzheimer’s Disease Data

**DOI:** 10.1038/s41598-019-55818-z

**Published:** 2019-12-20

**Authors:** Suprateek Kundu, Joshua Lukemire, Yikai Wang, Ying Guo, The Alzheimer’s Disease Neuroimaging Initiative, Michael W. Weiner, Norbert Schuff, Howard J. Rosen, Bruce L. Miller, Thomas Neylan, Jacqueline Hayes, Shannon Finley, Paul Aisen, Zaven Khachaturian, Ronald G. Thomas, Michael Donohue, Sarah Walter, Devon Gessert, Tamie Sather, Gus Jiminez, Leon Thal, James Brewer, Helen Vanderswag, Adam Fleisher, Melissa Davis, Rosemary Morrison, Ronald Petersen, Clifford R. Jack, Matthew Bernstein, Bret Borowski, Jeff Gunter, Matt Senjem, Prashanthi Vemuri, David Jones, Kejal Kantarci, Chad Ward, Sara S. Mason, Colleen S. Albers, David Knopman, Kris Johnson, William Jagust, Susan Landau, John Q. Trojanowki, Leslie M. Shaw, Virginia Lee, Magdalena Korecka, Michal Figurski, Steven E. Arnold, Jason H. Karlawish, David Wolk, Arthur W. Toga, Karen Crawford, Scott Neu, Lon S. Schneider, Sonia Pawluczyk, Mauricio Beccera, Liberty Teodoro, Bryan M. Spann, Laurel Beckett, Danielle Harvey, Evan Fletcher, Owen Carmichael, John Olichney, Charles DeCarli, Robert C. Green, Reisa A. Sperling, Keith A. Johnson, Gad Marshall, Meghan Frey, Barton Lane, Allyson Rosen, Jared Tinklenberg, Andrew J. Saykin, Tatiana M. Foroud, Li Shen, Kelley Faber, Sungeun Kim, Kwangsik Nho, Martin R. Farlow, AnnMarie Hake, Brandy R. Matthews, Scott Herring, Cynthia Hunt, John Morris, Marc Raichle, Davie Holtzman, Nigel J. Cairns, Erin Householder, Lisa Taylor-Reinwald, Beau Ances, Maria Carroll, Sue Leon, Mark A. Mintun, Stacy Schneider, Angela Oliver, Lisa Raudin, Greg Sorensen, Lew Kuller, Chet Mathis, Oscar L. Lopez, MaryAnn Oakley, Steven Paul, Norman Relkin, Gloria Chaing, Lisa Raudin, Peter Davies, Howard Fillit, Franz Hefti, M. Marcel Mesulam, Diana Kerwin, Marek-Marsel Mesulam, Kristine Lipowski, Chuang-Kuo Wu, Nancy Johnson, Jordan Grafman, William Potter, Peter Snyder, Adam Schwartz, Tom Montine, Elaine R. Peskind, Nick Fox, Paul Thompson, Liana Apostolova, Kathleen Tingus, Ellen Woo, Daniel H. S. Silverman, Po H. Lu, George Bartzokis, Robert A. Koeppe, Judith L. Heidebrink, Joanne L. Lord, Steven G. Potkin, Adrian Preda, Dana Nguyenv, Norm Foster, Eric M. Reiman, Kewei Chen, Adam Fleisher, Pierre Tariot, Stephanie Reeder, Steven Potkin, Ruth A. Mulnard, Gaby Thai, Catherine Mc-Adams-Ortiz, Neil Buckholtz, John Hsiao, Marylyn Albert, Marilyn Albert, Chiadi Onyike, Daniel D’Agostino, Stephanie Kielb, Donna M. Simpson, Richard Frank, Jeffrey Kaye, Joseph Quinn, Betty Lind, Raina Carter, Sara Dolen, Rachelle S. Doody, Javier Villanueva-Meyer, Munir Chowdhury, Susan Rountree, Mimi Dang, Yaakov Stern, Lawrence S. Honig, Karen L. Bell, Daniel Marson, Randall Griffith, David Clark, David Geldmacher, John Brockington, Erik Roberson, Hillel Grossman, Effie Mitsis, Leyla de Toledo-Morrell, Raj C. Shah, Debra Fleischman, Konstantinos Arfanakis, Ranjan Duara, Daniel Varon, Maria T. Greig, Peggy Roberts, James E. Galvin, Brittany Cerbone, Christina A. Michel, Henry Rusinek, Mony J. de Leon, Lidia Glodzik, Susan De Santi, P. Murali Doraiswamy, Jeffrey R. Petrella, Terence Z. Wong, Olga James, Charles D. Smith, Greg Jicha, Peter Hardy, Partha Sinha, Elizabeth Oates, Gary Conrad, Anton P. Porsteinsson, Bonnie S. Goldstein, Kim Martin, Kelly M. Makino, M. Saleem Ismail, Connie Brand, Kyle Womack, Dana Mathews, Mary Quiceno, Ramon Diaz-Arrastia, Richard King, Myron Weiner, Kristen Martin-Cook, Michael DeVous, Allan I. Levey, James J. Lah, Janet S. Cellar, Jeffrey M. Burns, Heather S. Anderson, Russell H. Swerdlow, Neill R. Graff-Radford, Francine Parfitt, Tracy Kendall, Heather Johnson, Christopher H. van Dyck, Richard E. Carson, Martha G. MacAvoy, Howard Chertkow, Howard Bergman, Chris Hosein, Sandra Black, Bojana Stefanovic, Curtis Caldwell, Ging-Yuek Robin Hsiung, Howard Feldman, Benita Mudge, Michele Assaly, Andrew Kertesz, John Rogers, Charles Bernick, Donna Munic, Andrew Kertesz, Andrew Kertesz, John Rogers, Elizabether Finger, Stephen Pasternak, Irina Rachinsky, Dick Drost, Carl Sadowsky, Walter Martinez, Teresa Villena, Raymond Scott Turner, Kathleen Johnson, Brigid Reynolds, Marwan N. Sabbagh, Christine M. Belden, Sandra A. Jacobson, Sherye A. Sirrel, Neil Kowall, Ronald Killiany, Andrew E. Budson, Alexander Norbash, Patricia Lynn Johnson, Joanne Allard, Alan Lerner, Paula Ogrocki, Leon Hudson, Smita Kittur, Michael Borrie, T-Y Lee, Rob Bartha, Sterling Johnson, Sanjay Asthana, Cynthia M. Carlsson, J. Jay Fruehling, Sandra Harding, Vernice Bates, Horacio Capote, Michelle Rainka, Douglas W. Scharre, Maria Kataki, Anahita Adeli, Eric C. Petrie, Gail Li, Earl A. Zimmerman, Dzintra Celmins, Alice D. Brown, Godfrey D. Pearlson, Karen Blank, Karen Anderson, Robert B. Santulli, Tamar J. Kitzmiller, Eben S. Schwartz, Kaycee M. Sink, Jeff D. Williamson, Pradeep Garg, Franklin Watkins, Brian R. Ott, Henry Querfurth, Geoffrey Tremont, Stephen Salloway, Paul Malloy, Stephen Correia, Jacobo Mintzer, Kenneth Spicer, David Bachman, Dino Massoglia, Nunzio Pomara, Raymundo Hernando, Antero Sarrael, Susan K. Schultz, Laura L. Boles Ponto, Hyungsub Shim, Karen Elizabeth Smith, Amanda Smith, Kristin Fargher, Balebail Ashok Raj, Karl Friedl, Jerome A. Yesavage, Joy L. Taylor, Ansgar J. Furst

**Affiliations:** 1 Department of Biostatistics and Bioinformatics, Emory University, Atlanta, Ga 30322 USA; 2 UC San Francisco, California, USA; 3 UC San Diego, California, USA; 4 Mayo Clinic, Rochester, New York USA; 5 UC Berkeley, California, USA; 6 UPenn, Philadelphia, Pennsylvania USA; 7 USC, Los Angeles, California, USA; 8 UC Davis, California, USA; 9 Brigham and Women’s Hospital/Harvard Medical School, Boston, Massachusetts USA; 10 Indiana University, Bloomington, Indiana USA; 11 Washington University St Louis, Missouri, USA; 12 Prevent Alzheimer’s Disease, 2020 Rockville, Maryland USA; 13 Siemens, Munich, Germany; 14 University of Pittsburg, Pennsylvania, USA; 15 Cornell University, Ithaca, New York USA; 16 Albert Einstein College of Medicine of Yeshiva University, Bronx, New York USA; 17 AD Drug Discovery Foundation, New York City, New York, USA; 18 Acumen Pharmaceuticals, Livermore, California USA; 19 Northwestern University, Evanston and Chicago, Illinois, USA; 20 National Institute of Mental Health, Rockville, Maryland USA; 21 Brown University, Providence, Rhode Island USA; 22 Eli Lilly, Indianapolis, Indiana, USA; 23 University of Washington, Seattle, Washington USA; 24 University of London, London, England; 25 UCLA, Los Angeles, California, USA; 26 University of Michigan, Ann Arbor, Michigan USA; 27 University of Utah, Salt Lake, Utah USA; 28 Banner Alzheimer’s Institute, Phoenix, Arizona USA; 29 UC Irvine, Irvine, California USA; 30 National Institute on Aging, Bethesda, Maryland USA; 31 Johns Hopkins University, Baltimore, Maryland USA; 32 Richard Frank Consulting, Washington, DC USA; 33 Oregon Health and Science University, Portland, Oregon USA; 34 Baylor College of Medicine, Houston, Texas USA; 35 University of Alabama, Birmingham, Alabama USA; 36 Mount Sinai School of Medicine, New York City, New York, USA; 37 Rush University Medical Center, Chicago, Illinois USA; 38 Wien Center, Miami, Florida USA; 39 New York University, New York City, New York, USA; 40 Duke University Medical Center, Durham, North Carolina USA; 41 University of Kentucky, Lexington, Kentucky USA; 42 University of Rochester Medical Center, Rochester, New York USA; 43 University of Texas Southwestern Medical School, Dallas, Texas USA; 44 Emory University, Atlanta, Georgia USA; 45 University of Kansas, Medical Center, Kansas City, Kansas USA; 46 Mayo Clinic, Jacksonville, Florida USA; 47 Yale University School of Medicine, New Haven, Connecticut USA; 48 McGill University/Montreal-Jewish General Hospital, Montreal, Quebec Canada; 49 Sunnybrook Health Sciences, Toronto, Ontario Canada; 50 U.B.C. Clinic for AD & Related Disorders, Vancouver, British Columbia Canada; 51 Cognitive Neurology–St Joseph’s, London, Ontario, Canada; 52 Cleveland Clinic Lou Ruvo Center for Brain Health, Las Vegas, Nevada USA; 53 St Joseph’s Health Care, London, Ontario, Canada; 54 Premiere Research Institute, Palm Beach Neurology, Miami, Florida USA; 55 Georgetown University Medical Center, Washington, DC USA; 56 Banner Sun Health Research Institute, Sun City, Arizona USA; 57 Boston University, Boston, Massachusetts, USA; 58 Howard University, Washington, DC USA; 59 Case Western Reserve University, Cleveland, Ohio USA; 60 Neurological Care of CNY, Liverpool, New York USA; 61 Parkwood Hospital, London, Ontario, USA; 62 University of Wisconsin, Madison, Wisconsin USA; 63 Dent Neurologic Institute, Amherst, New York USA; 64 Ohio State University, Columbus, Ohio USA; 65 Albany Medical College, Albany, New York USA; 66 Hartford Hospital, Olin Neuropsychiatry Research Center, Hartford, Connecticut USA; 67 Dartmouth-Hitchcock Medical Center, Lebanon, New Hampshire USA; 68 Wake Forest University Health Sciences, Winston-Salem, North Carolina USA; 69 Rhode Island Hospital, Providence, Rhode Island USA; 70 Butler Hospital, Providence, Rhode Island USA; 71 Medical University South Carolina, Charleston, South Carolina USA; 72 Nathan Kline Institute, Orangeburg, New York USA; 73 University of Iowa College of Medicine, Iowa City, Iowa USA; 74 University of South Florida: USF Health Byrd Alzheimer’s Institute, Tampa, Florida USA; 75 Department of Defense, Arlington, Virginia USA; 76 Stanford University, Stanford, California, USA

**Keywords:** Computational neuroscience, Neurological disorders

## Abstract

There is well-documented evidence of brain network differences between individuals with Alzheimer’s disease (AD) and healthy controls (HC). To date, imaging studies investigating brain networks in these populations have typically been cross-sectional, and the reproducibility of such findings is somewhat unclear. In a novel study, we use the longitudinal ADNI data on the whole brain to jointly compute the brain network at baseline and one-year using a state of the art approach that pools information across both time points to yield distinct visit-specific networks for the AD and HC cohorts, resulting in more accurate inferences. We perform a multiscale comparison of the AD and HC networks in terms of global network metrics as well as at the more granular level of resting state networks defined under a whole brain parcellation. Our analysis illustrates a decrease in small-worldedness in the AD group at both the time points and also identifies more local network features and hub nodes that are disrupted due to the progression of AD. We also obtain high reproducibility of the HC network across visits. On the other hand, a separate estimation of the networks at each visit using standard graphical approaches reveals fewer meaningful differences and lower reproducibility.

## Introduction

Alzheimer’s disease (AD) is one of the most common forms of dementia. An increasing emphasis is being placed on identifying biomarkers for the detection of AD at an early or pre-clinical stage in hopes of limiting the neuronal damage caused by AD. One such promising biomarker is disruptions in healthy patterns of resting state functional connectivity, which can be detected using resting state functional magnetic resonance imaging (rsfMRI)^1–3^. The National Institute on Aging–Alzheimer’s Association lists rsfMRI functional connectivity as a potential biomarker of neuronal injury, at an early stage of validation^4^.

One of the most popular approaches to investigating functional connectivity is using brain networks defined through a graph-theoretic approach that defines the brain network as a set of connections (known as edges) between brain regions (commonly called nodes). A graph or network ${\mathscr G}$ is characterized by its node set ${\mathscr V}$ and edge set ${\mathscr E}$. Given the node set ${\mathscr V}=\{1,\ldots ,p\}$, the goal of a graph-theoretic approach for computing functional connectivity is to estimate the edge set ${\mathscr E}$ using the fMRI data. Graph-theoretical studies of functional connectivity in HC and AD patients typically estimate the brain network separately for each individual or cohort and then examine differences in these networks to identify brain regions with disrupted connectivity in AD patients^5,6^. These differences can be investigated at different levels of granularity, such as at the edge level, node level, or at the level of global or local network metrics.

To date, graph theoretical studies of the brain network in HC and AD have been predominantly cross-sectional. However in recent years longitudinal studies have become increasingly popular in the neuroscience community. By collecting baseline and follow-up data for each subject, longitudinal studies are well-known to have the potential to provide more reliable and significant scientific findings than cross-sectional studies. There has been some limited work on longitudinal analysis of brain connectivity, which mainly involves modeling pairwise connectivity measures or network summary measures from a pre-specified network structure^7–9^. Very recently, a small number of studies^10–13^ looked at longitudinal modifications in the AD network and compared these to network changes for HC and/or MCI groups. Compared to cross-sectional data, a key advantage in longitudinal fMRI studies is that one can potentially use state of the art statistical methods to jointly estimate the brain networks over multiple visits by pooling information across visits. Joint estimation of multiple brain networks has recently been shown to result in greater power to detect the true edges and significant differences, compared to the commonly used approach of separately estimating each network^14^. However, to our knowledge none of the existing AD studies have pooled information across visits to estimate cohort- and visit-specific networks. Moreover, few existing approaches used sparse precision (or inverse covariance) matrices, which provides a nice graph theoretic interpretation and is the focus of this paper. Such matrices encode conditional dependency relationships between measurements with off-diagonal zeros corresponding to absent edges.

The standard practice for comparing multiple brain networks is to estimate each brain network separately and then use mass-univariate hypothesis testing to infer significant differences between the networks while controlling the family-wise error rate^15–17^. These approaches suffer from inflated false positive rates because of the massive number of multiple comparisons they use^18^, and they also possibly have reduced power to detect true differences between visits^19^. As an alternative to these large scale hypothesis testing procedures, network metrics and statistics have become quite popular for summarizing the large scale organization of the brain^18–20^. These techniques improve statistical power but come at the cost of reduced explanatory value since they typically do not provide inferences at the level of individual connections.

In order to take advantage of the benefits of pooling data across cohorts/visits for network estimation, there has been a recent growth in penalized approaches for the joint estimation of multiple graphical models. These include penalized approaches^21–23^ that only report point estimates and do not provide measures of uncertainty, and some Bayesian methods as in^24^ and^25^ that provide a convenient way to quantify uncertainty. However with the exception of some very recent work^26^, none of the existing approaches for the joint estimation of multiple networks have been applied to the problem on estimating multiple temporally dependent networks across longitudinal visits, to our knowledge. Unfortunately, the approach by^26^ uses a regression based model that is unable to produce a positive definite precision matrix which is key to quantifying the edge strengths.

Our study is motivated by the longitudinal ADNI data, which contains rsfMRI measurements for each individual (belonging to HC or AD cohorts) at baseline and follow-up visits. Using this data, one can compute and compare the brain networks for the AD and HC cohorts at each visit by pooling information across visits using graph theoretic approaches. While some limited variations are expected in both networks due to the inherent variability in the data, we expect to see systematic longitudinal differences in the AD network between baseline and the follow-up visits due to the progression of the disease. Network differences between cohorts that are consistent across visits, as well as similarity of the HC network between visits, would be suggestive of reproducible findings that are robust to noise inherent in the fMRI data. Our focus in this work is on (i) inferring network changes between the two cohorts at each visit; (ii) evaluating whether such network differences between cohorts are consistent across the two visits, and (iii) understanding the longitudinal network changes for the AD individuals and assessing network reproducibility for the HC cohort across visits. We would also like to compare the above findings in relation to the results obtained under an alternate analysis involving the separate estimation of the network for each cohort and visit using standard graphical modeling approaches. Some key contributions of this article are:

- We propose a graph theoretic analysis of the network changes in AD individuals compared to the HC network at baseline and one-year follow-up using longitudinal ADNI rsfMRI data.
- We use the cutting edge Bayesian joint network learning (BJNL) approach^14^ to jointly estimate the brain networks at the two visits for each cohort. The joint learning is able to detect a better separation between the AD and HC networks and leads to more reproducible estimates for the HC network. In contrast, a separate estimation of each cohort- and visit-specific network suggests a lack of separation of the HC and AD networks and poor reproducibility of the HC network across visits.
- Our analysis provides a multiscale understanding of the network changes between AD and HC networks, at the level of global network metrics, as well for the more local resting state networks and hub nodes. We illustrate the loss of small-world properties as well as the disruption of other global network metrics in the AD network, and identify the hub nodes responsible for the greatest disruptions.

Our analysis results make a strong case for the merits of joint estimation of longitudinal networks by pooling data across visits. To our knowledge, this work is one of the first such studies to illustrate the benefits of joint analysis in brain network estimation using longitudinal neuroimaging data.

## Methods

### Description of the data

#### Rs-fMRI description

We applied the BJNL method to the longitudinal rsfMRI data from the Alzheimer’s Disease Neuroimaging Initiative 2 (ADNI2) study. One of the main purposes of the ADNI2 project is to examine changes in neurobiology with the progression of AD. Data used in our analysis were downloaded from the ADNI website (http://www.adni.loni.usc.edu) and included longitudinal rs-fMRI images that were collected at baseline screening and 1 year follow-up, for 17 individuals with AD, and 27 healthy controls (HC). Although data was also available for a second follow-up visit at year 2, we limit our analysis to scans from baseline and one-year, since the sample size for individuals with data at all three visits was fairly small. More detailed descriptions of the demographic and clinical features of the participants are provided in Table 1. The acquisition protocol and pre-processing steps for the imaging data are described in the Supplementary Materials.

**Table 1:** Demographics table for the healthy controls (HC), early MCI (EMCI), late MCI (LMCI), and Alzheimer’s disease patients (AD).

N	HC	EMCI	LMCI	AD
	27	24	23	17
Baseline Age (sd)	74.02 (5.88)	71.62 (5.66)	71.38 (7.21)	73.91 (8.48)
Sex (% Female)	55.6	37.5	43.5	58.8
Education (sd)	16.26 (2.12)	15.96 (2.46)	16.48 (2.45)	14.88 (2.62)
APOE4 (number with)				
0	17	12	15	2
1	9	9	5	9
2	1	3	3	6
Amyloid (% Positive)	26.9	58.3	60.9	100

One HC individual had missing amyloid status.

We note that the sample size used for our analysis is comparable to other recent longitudinal analyses using ADNI data, although none of the existing studies have looked at longitudinal brain network changes using resting state fMRI data jointly from multiple visits. For example^27^, analyzed resting state fMRI data from ADNI involving 30 healthy controls (17 females and 13 males) and 26 AD patients (11 females and 15 males). Also^9^ performed a longitudinal analysis of resting state connectivity using data from ADNI 2 with 10 AD and 23 HC individuals^13^. proposed a longitudinal independent component analysis using an even smaller sample size from ADNI 2 study. In another study involving 40 participants (5 AD, 16 MCI and 19 HC)^28^, used longitudinal PET data at baseline and two year followup from ADNI GO and ADNI 2 databases, to study changes in white matter hyperintensities. As in the above studies, the moderate sample size for our analysis is sufficient to detect important network differences between the AD and HC cohorts.

#### Power atlas and resting state networks

The preprocessed fMRI data was summarized into the 264 node system specified by the Power atlas in^29^. The Power atlas^29^ is based on functional systems of brain activity identified using task and resting-state fMRI. As a further level of parcellation, each node was assigned to one of 10 resting state networks (RSNs) identified in^30^ using the technique described in^31^. The RSNs used and number of nodes in each RSN (in parenthesis) were as follows: medial visual network (15), occipital pole visual network (15), lateral visual network (19), default mode network (20), cerebellum (6), sensorimotor (31), auditory (29), executive control (39), right frontoparietal (32), left frontoparietal (26), and the remainder of the nodes were assigned as unknown (32). We also note that the Cerebellum has an extremely small number of nodes, which may potentially result in extreme (and potentially unreliable) values of some network metrics as seen from the results presented in the article.

### Bayesian joint network learning

Recently^14^, proposed the BJNL approach to jointly estimate multiple networks under a Bayesian framework. BJNL models the edge probabilities as a flexible function of shared components that are common across all networks and differential components unique to each specific network. The approach provides a convenient framework to pool information across multiple networks to model edges without enforcing similarity of the edge strengths across networks. It is automatically able to provide measures of uncertainty and yield positive definite precision matrix estimates that can be used to systematically quantify edge strengths. In their paper^14^, showed the BJNL approach could result in substantial improvements for the estimation of multiple networks corresponding to different cognitive states in a visual experiment, compared to other existing approaches that estimate multiple networks jointly or separately. In this article, we use the BJNL approach for analyzing the longitudinal ADNI data, in order to jointly estimate cohort- and visit-specific resting state brain networks corresponding to the baseline and one-year follow-up visits for AD individuals and separately for HC subjects.

The BJNL approach relies on a Gaussian graphical model (GGM) to fit the brain functional network based on the fMRI measurements which are assigned a Gaussian distribution characterized by a sparse inverse covariance matrix. That is, the prewhitened fMRI measurements **y** are modeled: ${\bf{y}} \sim N({\bf{0}},{\Omega }_{{\mathscr G}}^{-1})$, where the off-diagonal elements of Ω are assigned zeros corresponding to absent edges in the network ${\mathscr G}$. As in^14^, we use a autoregressive model to prewhiten the fMRI measurements (i.e. minimize temporal correlations) in order to satisfy the independence assumption typically required for GGMs. The goal of a GGM is to estimate a sparse precision matrix from the data, which automatically enables one to infer the estimated network $\hat{{\mathscr G}}$ simply by examining which elements of the precision matrix are non-zero. The BJNL approach that we are going to use for our analysis is based on the joint estimation of multiple GGMs. The parameters are sampled iteratively using Markov chain Monte Carlo (MCMC), and the MCMC samples are aggregated to obtain parameter estimates and report credible intervals that quantify uncertainty. An overview of the BJNL method is provided in the Supplementary Materials; we refer the reader to^14^ for full details.

One potential pitfall of our approach is that it views the data at the group level, which does not account for subject-level heterogeneity in the brain networks. A possible approach for accounting for heterogeneity is to model individual specific networks as a function of covariates, that will also enable one to pool information across subjects when estimating the covariate effects on the networks. However, such an approach is methodologically challenging, and to our knowledge, most of the existing methods for joint estimation of Gausssian graphical models can not directly incorporate covariate information when estimating multiple networks. Moreover, our focus in this article is on group level comparisons, and we expect the effects of covariates such as age, sex, and education, on the cohort level network differences to balance out (by design) when comparing the AD and HC groups having similar distributions of the covariates. Hence, although we did not explicitly account for age, sex, and education when modeling the networks, these variables are not expected to drive any important differences between the AD and HC networks, since the composition of the cohorts with respect to the covariates are similar.

### Graph metrics and small world organization

We use the BJNL approach to pool data across visits in order to compute visit-specific networks for the AD and HC cohorts. Using these computed networks, we perform a multi-scale analysis of the differences in the AD and HC networks at baseline and one-year follow-up using network metrics at the global network level, as well as at the level of RSNs and nodes. These metrics were computed using the Brain Connectivity Toolbox^32^, and allow us to use single numbers to summarize efficiency of information transmission and other aspects of brain network organization such as small-worldedness and clustering. For our analysis, we focused on global efficiency (GE), local efficiency (LE), characteristic path length (CPL), mean clustering coefficient (MCC), and small-worldedness, which were calculated based on the estimated adjacency matrices from BJNL. Adjacency matrices refer to binarized matrices with off-diagonal ones corresponding to edges in the network and off-diagonal zeros otherwise. A description of the metrics used is provided in the Supplementary Materials.

We note that the CPL and GE metrics are inversely proportional (i.e. a higher GE implies smaller CPL and vice-versa), and both these metrics are related to the small-worldedness of the network. Our goal is to use the previously defined metrics to investigate the disruption of the AD network compared to the HC network at baseline and one-year follow-up, and to identify the nodes and RSNs responsible for the greatest disruptions. In particular, we also examine some RSN-specific metrics that help us identify which RSNs result in the greatest differences in brain organization between the AD and HC networks at baseline and one-year follow-up. On the other hand, a node attack approach is used to detect nodes with the highest disruptive potential (see below). These comparisons are designed to provide a multiscale understanding of the network disruptions in AD and how it modifies information transmission in the brain.

#### Metrics for identification of hub nodes

Hub nodes are generally identified by how often a node appears in the shortest path of other nodes or by how many of a node’s connections are to nodes in RSNs other than the RSN to which that node belongs. We used the Brain Connectivity Toolbox to calculate the participation coefficient and betweenness (described in the Supplementary Materials), both of which are node-specific metrics that can identify hub nodes.

### Node attack approach

To study the node-specific contributions to information transmission, we use a node attack approach^33,34^ where we cycle through each node in the brain and remove all edges to that node, effectively disconnecting it from the brain network. Then, we recalculate the global network metrics on the resulting network and identify the important nodes as those responsible for the maximum disruptions when they are disconnected from the network. In a small-world network, deletion of a randomly selected node is not expected to have much influence on most network metrics due to the fact that most information transmission runs through a small number of hub nodes. On the other hand, if the deleted node is a hub node, then we expect to see a significant changes in the network metrics. The nodes resulting in the highest reductions in the metrics were identified and listed in Tables 2–4.

**Table 2:** Top 10 largest percent change in global efficiency upon removal of a node in HC and AD.

Node	RSN	HC Baseline	HC One-year	AD Baseline	AD One-year	*AD* − *HC* Baseline	*AD* − *HC* One-year
85	SM	−1.04	−1.16	−0.85	−0.83	0.18	0.32
81	SM	−1.00	−1.08	−0.91	−0.86	0.09	0.21
51	DMN	−1.06	−0.96	−0.88	−0.86	0.19	0.09
64	DMN	−0.96	−1.06	−0.94	−0.85	0.03	0.2
137	EC	−0.97	−1.02	−0.83	−0.82	0.14	0.2
166	EC	−0.98	−0.88	−0.82	−0.78	0.16	0.11
59	DMN	−0.98	−0.98	−0.79	−0.81	0.18	0.17
143	EC	−0.97	−0.94	−0.85	−0.82	0.12	0.12
70	Cerebellum	−0.95	−0.84	−0.77	−0.77	0.18	0.07
71	Cerebellum	−0.95	−0.85	−0.81	−0.83	0.13	0.02

The final two columns display the difference in percent loss between AD and HC at that node at baseline and at one−year. All of the differences between AD and HC are positive, indicating that HC saw a larger reduction in global efficiency.

**Table 3:** Top 10 largest percent change in CPL upon removal of a node in HC and AD.

Node	RSN	HC Baseline	HC One-year	AD Baseline	AD One-year	*AD* − *HC* Baseline	*AD* − *HC* One-year
85	SM	0.37	0.54	0.1	0.07	−0.28	−0.47
81	SM	0.34	0.44	0.17	0.09	−0.17	−0.35
51	DMN	0.44	0.29	0.14	0.11	−0.3	−0.18
64	DMN	0.29	0.41	0.24	0.1	−0.05	−0.31
137	EC	0.29	0.34	0.08	0.07	−0.21	−0.27
59	DMN	0.3	0.28	0.05	0.06	−0.25	−0.22
166	EC	0.29	0.17	0.05	0.02	−0.24	−0.15
143	EC	0.29	0.23	0.09	0.06	−0.2	−0.17
30	Occ Pole	0.27	0.18	0.04	0.03	−0.23	−0.15
71	Cerebellum	0.26	0.13	0.06	0.07	−0.2	−0.06

Positive (negative) values indicate that removal of this node increased (decreased) the CPL. The final two columns display the difference in percent change between AD and HC at that node at baseline and at one-year.

**Table 4:** Top 10 largest percent change in MCC upon removal of a node in HC and AD.

Node	RSN	HC Baseline	HC One-year	AD Baseline	AD One-year	AD − HC Baseline	AD − HC One-year
*211	FPR	−0.5	−0.77	−0.58	−0.52	−0.08	0.25
*27	Occ Pole	−0.75	−0.49	−0.25	−0.43	0.5	0.06
*236	Unk	−0.39	−0.74	−0.53	−0.47	−0.13	0.27
*228	FPR	−0.55	−0.57	−0.7	−0.45	−0.15	0.12
81	SM	−0.21	−0.33	−0.69	−0.66	−0.48	−0.34
210	FPR	−0.37	−0.64	−0.56	−0.69	−0.19	−0.05
*195	FPL	−0.67	−0.69	−0.69	−0.67	−0.01	0.01
183	FPL	−0.45	−0.33	−0.68	−0.51	−0.23	−0.17
208	FPR	−0.27	−0.68	−0.42	−0.51	−0.15	0.18
191	FPL	−0.44	−0.31	−0.53	−0.65	−0.09	−0.34

Positive (negative) values indicate that removal of this node increased (decreased) the MCC. The final two columns display the difference in percent change between AD and HC at that node at baseline and at one-year. A * before a node indicates that it was not a hub in any of the cohorts/visits.

### Assessing statistically significant differences

The statistically significant differences between networks are assessed by constructing credible intervals for each of the quantities of interest and examining the 100(1 − *α*)% credible intervals obtained using MCMC sampling from the posterior distributions BJNL approach in the. An overlap of the credible intervals implies non-significant differences for the metrics, whereas no overlap implies significant variations. In order to adjust for the multiple comparisons (baseline vs. one-year for each metric (10) and HC vs. AD for each metric (5)), we use a multiplicity adjusted threshold *α* = 0.01/15 = 0.0008 due to Bonferroni correction. For testing differences in nodal metrics such as betweenness and participation coefficient, whose posterior distributions are not normal and the separation in the posterior distributions between cohorts is not clear, we use Wilcoxan signed-rank test that is a non-parametric test free of distributional assumptions.

### Reproducibility of the network

**Figure 1:**
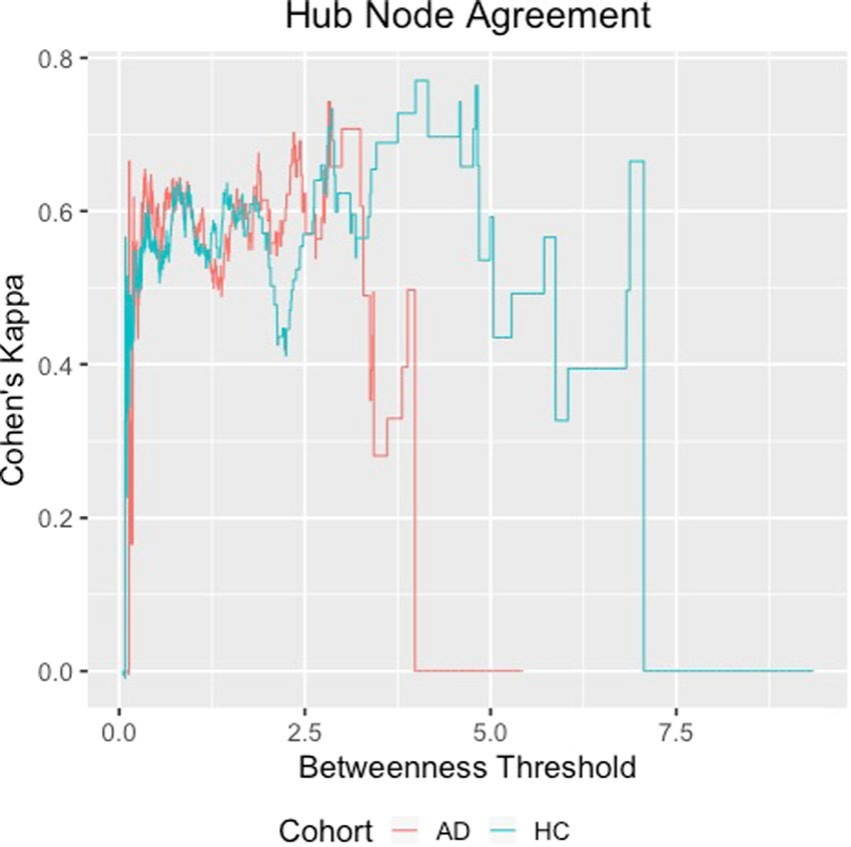
Kappa agreement statistics by normalized betweenness required to call a node a hub node for HC and AD.

In order to evaluate the overlap of the estimated AD and HC networks over the two visits (baseline and one-year follow-up), we investigated the consistency of hub nodes over time using Cohen’s kappa coefficient^35^. We defined hub nodes as nodes where the normalized betweenness score was 1.5 standard deviations above the mean^34,36^. We varied the threshold of normalized betweenness required to call a node a hub node from the minimum to the maximum value and calculated the kappa coefficient for each threshold. Bigger values of the threshold imply a smaller number of hub nodes having many connections, and larger values of the kappa coefficient indicate agreement across time points in what can be considered hub nodes. The results for the hub nodes are presented in Fig. 1. To obtain a more general assessment of agreement between networks across visits, we also computed the reproducibility for several network metrics across visits using a measure known as intraclass correlation coefficient or ICC^37^. Using the measure ICC, we investigate the reliability of graph metrics across two scanning sessions^38,39^. This metric is commonly used to measure test-retest network stability in brain networks^37^ with agreement scale 0 < *ICC* ≤ 0.2(slight), 0.2 < *ICC* ≤ 0.4 (fair), 0.4 < *ICC* ≤ 0.6 (moderate), 0.6 < *ICC* ≤ 0.8 (strong), and 0.8 < *ICC* ≤ 1 (near perfect) as suggested by^39^.

ICC is usually derived by calculating the proportion of the total variation attributed to variability across scanning sessions. Thus, small variation across sessions produces high ICC values, indicating strong reproducibility. In network estimation problems for neuroimaging applications, one expects strong reproducibility across different scanning sessions for networks with no systematic changes between time points (such as in healthy controls), but such reproducibility is not expected in networks for diseased individuals where the progression of the disease (e.g. AD) is likely to alter the network for the same individual across visits.

## Results

### Estimated connectivity

**Figure 2:**
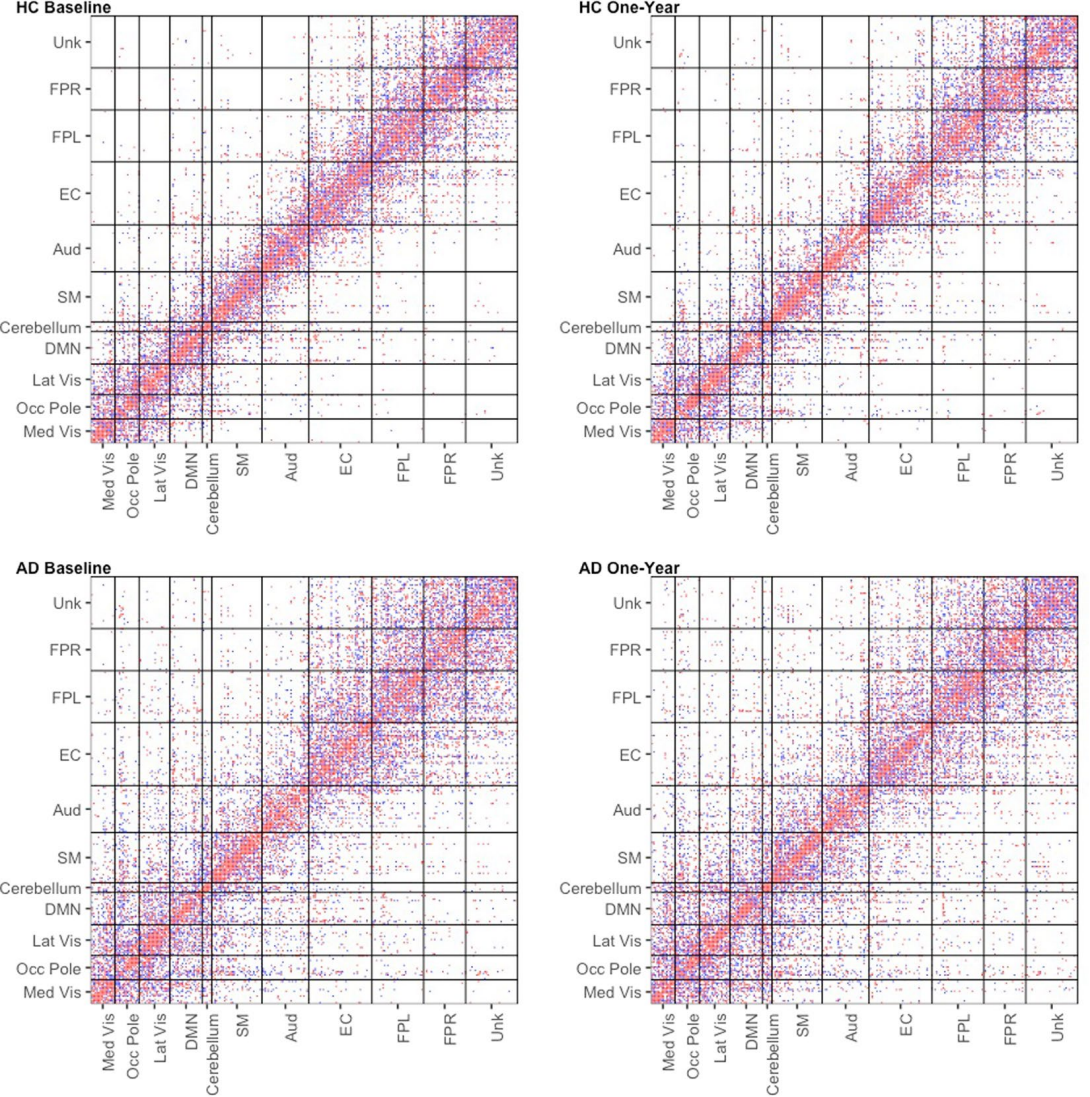
Estimated adjacency matrices for HC and AD at baseline and one-year follow-up. The edges are colored by the sign of the partial correlation (blue = negative, red = positive). There are 6444 and 6029 estimated edges in HC at baseline and one-year follow-up respectively. There are 8049 and 8401 estimated edges in AD at baseline and one-year follow-up respectively. Edges in AD network appear to be more randomly distributed compared to the HC network, which has more within RSN connections and fewer between RSN connections. The RSNs are abbreviated follows: medial visual network (Med vis), occipital pole visual network (Occ pole), lateral visual network (Lat vis), default mode network (DMN), cerebellum (Cerebellum), sensorimotor (SM), auditory (Aud), executive control (EC), right frontoparietal (FPR), left frontoparietal (FPL), and unknown (Unk).

The estimated adjacency matrices for HC and AD are presented in Fig. 2. The connections are colored based on the sign of the corresponding partial correlation. We identified 6444 and 6029 edges in HC at baseline and one-year follow-up respectively, that represent 18.6% and 17.4% of the possible connections in the network. We identified 8049 and 8401 edges in AD at baseline and one-year follow-up respectively, that represent 23.2% and 25.2% of the possible connections in the network. Hence the densities of the estimated networks are well within the range of network densities expected in practical fMRI applications^37^. From Fig. 2, it is fairly evident that the HC network has a smaller number of connections that are mostly concentrated within RSNs, while the AD network has a greater number of between-RSN connections and in general the AD network connections are more randomly distributed. In comparison to 40.1% and 40.0% connections that were contained within RSNs for the HC network at baseline and one-year, only 30.3% and 30.3% of the connections were contained within RSNs for the AD network at baseline and one-year.

### Graph metrics and small-worldedness

**Figure 3:**
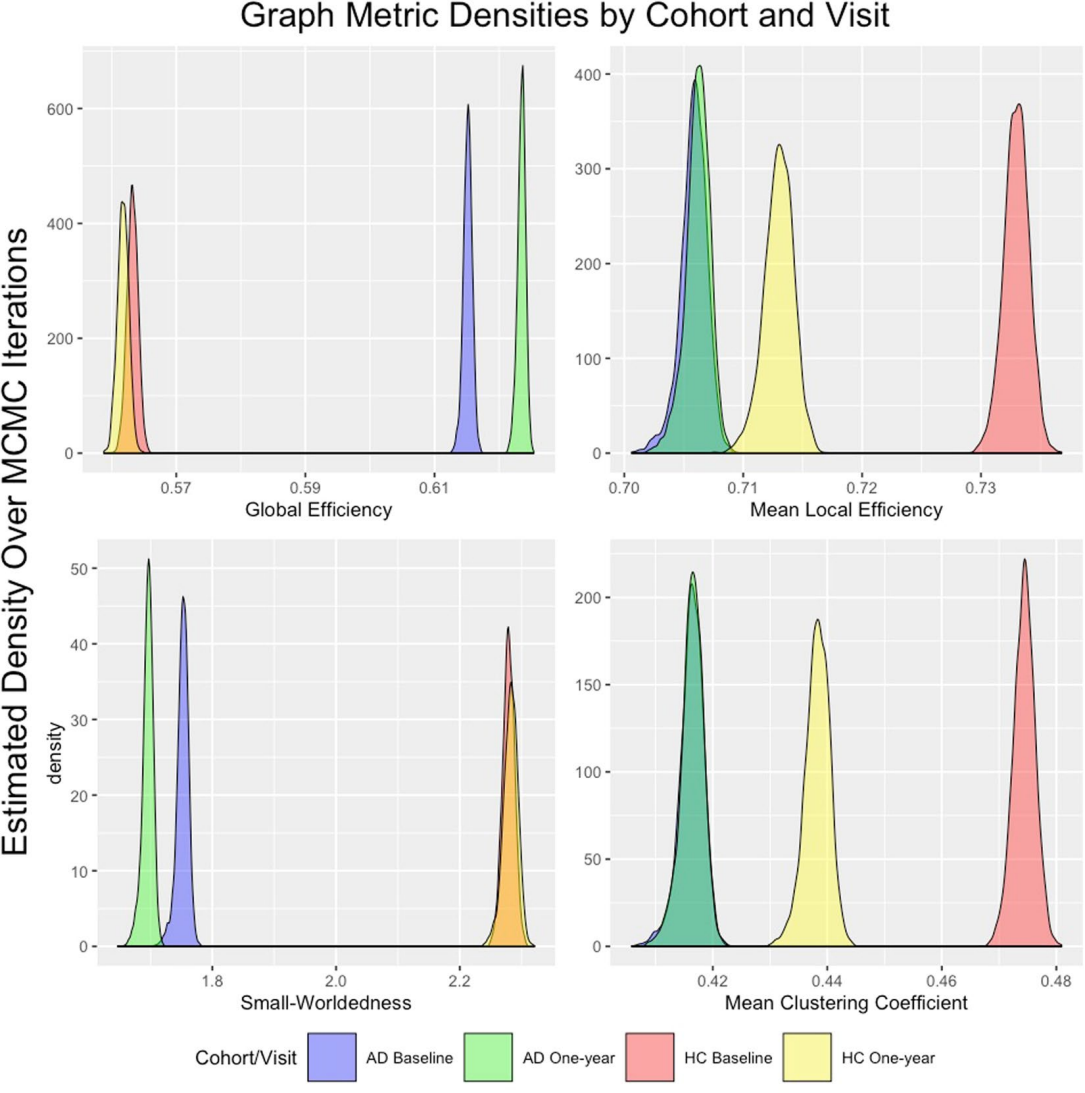
Graph metrics for each cohort as identified by BJNL.

The posterior densities for the graph metric results are presented in Fig. 3. There is no overlap in the estimated densities for the network metrics between the HC and AD groups both at baseline and one-year follow-up, with the exception of some minimal overlap in average local efficiency between the HC One-Year and AD groups. These results indicate significant differences between the AD and HC networks (*p* < 0.01 in all cases, Bonferroni corrected), which is somewhat expected based on evidence in existing literature.

These findings imply a greater clustering tendency and higher local efficiency at the node level in the HC network, compared to the AD network. The increased MCC for the HC network reflects the tendency of the connections to be clustered within RSNs (as evidenced by the adjacency matrix plot in Fig. 2). A greater MCC is also explained by the presence of a much larger number of hub nodes (Table 5), indicating the propensity of the edges to be clustered around a subset of important regions in the brain. Our findings in Fig. 3 also imply a greater small-world organization for the HC network, which is consistent with existing evidence in the literature^40,41^, and supports our hypothesis that the AD network is more closer to a random network compared to the HC network.

**Table 5:** Hubs in each cohort and visit identified using a normalized betweenness score 1.5 standard deviations above the mean organized by resting state network.

Cohort/Visit	#Hub	Med Vis	Occ Pole	Lat Vis	DMN	Cerebellum	SM	Aud	EC	FPL	FPR	Unk
HC - Baseline	20	0	2	0	5	2	2	1	4	2	2	0
HC - One-Year	23	0	4	0	4	0	3	2	5	3	2	0
AD - Baseline	14	0	3	0	2	0	3	2	4	0	0	0
AD - One-Year	14	0	2	0	2	1	4	3	2	0	0	0

**Figure 4:**
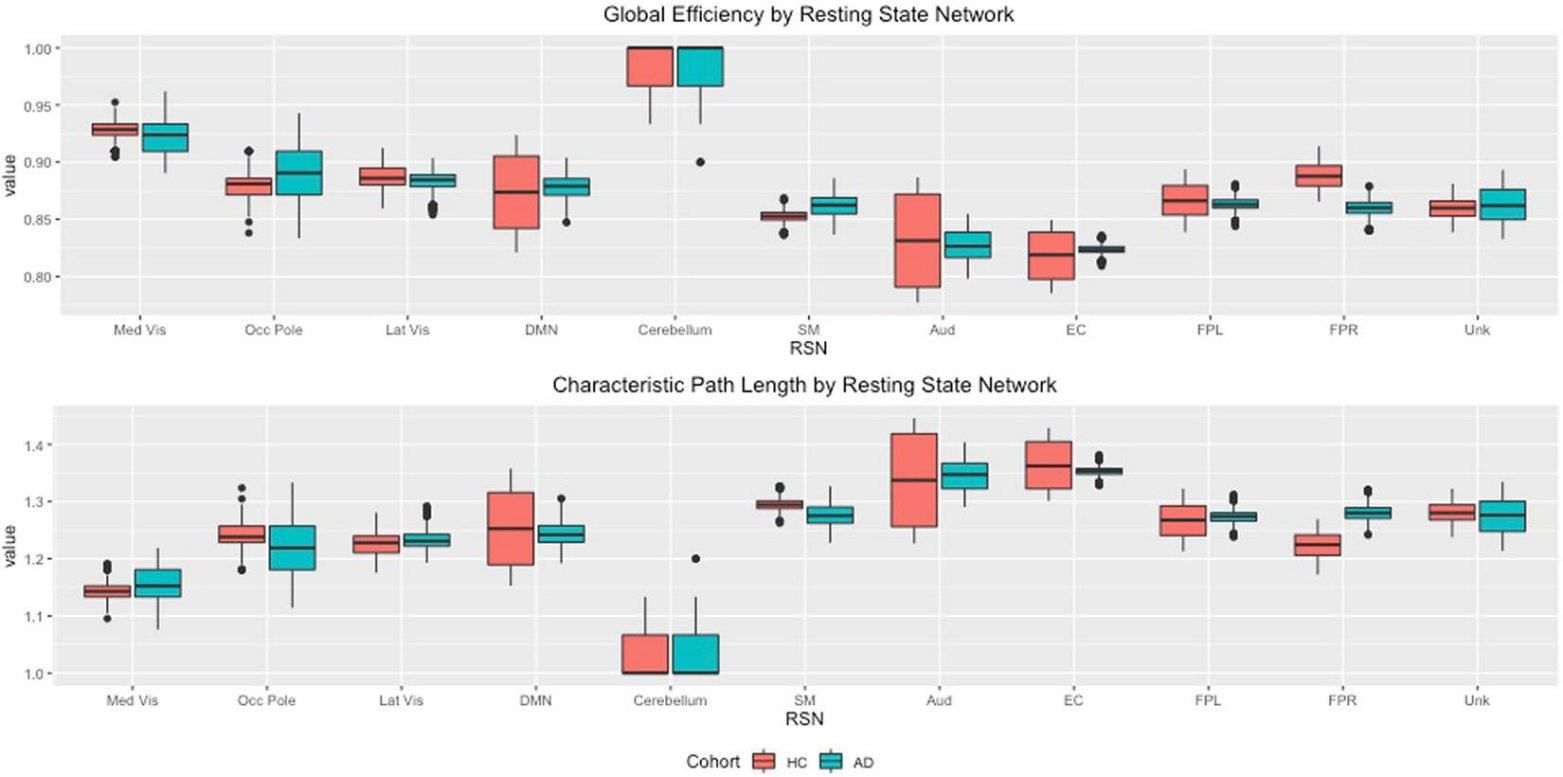
Boxplots of Global Efficiency and CPL within each resting-state network over MCMC iterations.

Surprisingly, the AD network seems to have a smaller CPL and larger GE at a global level, compared to the HC network. Both of these metrics measure the efficiency of information transmission, which is typically thought to be higher in the HC network compared to the AD network. In order to investigate the reason for this paradoxical result, we examined the GE and CPL values for each RSN in the AD and HC networks. Boxplots of the GE and CPL within each resting-state network are presented in Fig. 4 for each cohort averaged over visits. The results indicate that when looking at the metrics within RSN, the observed differences tend to largely go away except the FPR functional module in which the efficiency is significantly greater under the HC network. Our findings imply that the efficiency of information transmission in the HC network is largely similar within each RSN, and that the paradoxical findings in Fig. 3 can be attributed to a large number of between RSN connections under the AD network that are absent in the HC network (Fig. 2). We note that earlier findings have directly implicated the fronto-parietal regions in the progression of AD (see review by^42^).

Last, but not the least, the network metric differences between the AD and the HC networks are consistent across baseline and one-year follow-up. It is also of interest to note the much greater overlap of the global efficiency and small worldedness at baseline and one-year follow-up for the HC group, compared to the AD network, in Fig. 3. The overlap of the HC network across baseline and one-year follow-up is also reflected by the consistency of the hub nodes in Fig. 1, as identified using the betweenness coefficient. The Figure clearly illustrates the consistently greater agreement of the hub nodes under the HC networks across almost all thresholds for the betweenness coefficient, which implies greater similarity between the baseline and one-year HC networks, potentially due to the preservation of small-worldedness across visits. On the other hand, the AD network demonstrates increased separability across visits in terms of global efficiency and small worldedness in Fig. 3, as well as poor agreement of the hub nodes in Fig. 1, both of which are indicative of the progression of AD and the more random nature of the network.

### Reproducibility for the HC network

Under our analysis, the computed ICC values under the HC network at baseline and one-year follow-up for the betweenness coefficient, participation coefficient, nodal degree, local efficiency, and clustering coefficient were 0.80, 0.81, 0.82, 0.76, and 0.85, respectively. Clearly, the high ICC values over a range of local network metrics point towards a strong to near perfect reproducibilty of the HC network between baseline and the one-year follow-up visit, as would be expected in a healthy brain that is experiencing few connectivity changes over time. However, the high ICC values are also indicative of the robustness of the BJNL procedure, especially when contrasted with the considerably lower ICC values under an alternate analysis involving a separate estimation of the networks under the graphical lasso (see below).

### Role of hub nodes

**Figure 5:**
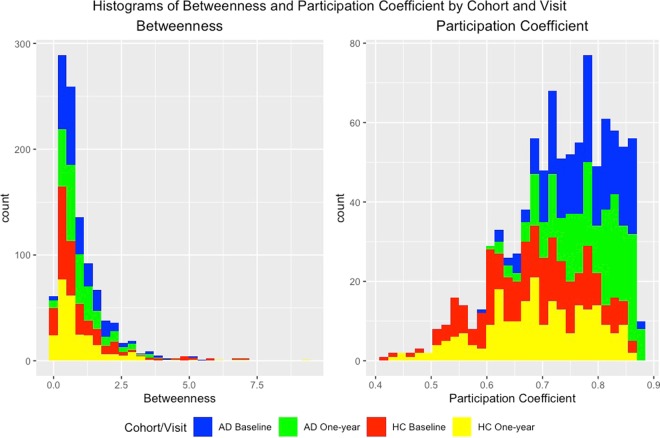
Histograms of the posterior mean for betweenness and participation coefficient across nodes. Note that only the 239 nodes belonging to a RSN were used to calculate the participation coefficient.

**Figure 6:**
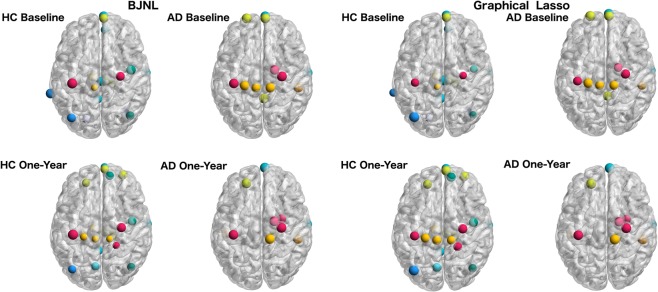
Hub nodes within the brain for each cohort and visit as identified using BJNL and the graphical lasso. The nodes are colored by resting state network.

Figure 5 displays histograms of the posterior mean normalized betweenness and participation coefficient for each node that were used to determine hub nodes. Because the distributions of the values across the nodes do not appear to be normally distributed, we use a Wilcoxan Signed-Rank test to test for significant differences between the two cohorts. We find significant differences in normalized betweenness (*p* = 1.48 × 10^−5^), and also for the participation coefficient (*p* < 2.2 × 10^−16^), between the HC and AD networks. These results support the findings presented in Table 5 and Fig. 2 that illustrate a greater number of hub nodes, and the smaller number of between RSN connections in the HC network. The spatial locations of the hub nodes are displayed in Fig. 6.

A list of all hub nodes identified by cohort and visit is provided in Table 6 and represented in Fig. 6. The highest number of hub nodes for the HC network were detected in the default mode network (DMN) and the executive control (EC). However, in the AD group a fewer number of hubs were identified in these RSNs, with the exception of the AD group at baseline, which still showed four hubs in the EC at baseline, but this count had reduced to two by one-year follow-up. Literature has suggested changes in DMN activity that distinguishes AD from a healthy aging cohort^43^, and differences in the EC network have also been identified^44^. Table 5 also illustrates that no hub nodes corresponded to the remaining nodes with an unallocated RSN, which validates the use of the pre-specified RSNs for our analysis.

**Table 6:** Hubs in each cohort and visit identified using a normalized betweenness score 1.5 standard deviations above the mean.

Cohort/Visit	Node Index of Hub Nodes
HC - Baseline	18, 30, 51, 52, 59, 64, 69, 70, 71, 81, 85, 112, 137, 143, 149, 166, 177, 181, 207, 212
HC - One-Year	18, 19, 20, 30, 51, 59, 64, 67, 81, 85, 88, 124, 135, 137, 141, 143, 164, 166, 177, 181, 184, 207, 210
AD - Baseline	18, 19, 20, 51, 64, 81, 85, 99, 132, 135, 137, 143, 149, 152
AD - One-Year	18, 19, 51, 64, 71, 81, 85, 99, 106, 118, 124, 132, 137, 141

The participation coefficient across all nodes were 0.684, 0.690, 0.775, 0.782 in HC Baseline, HC One-Year, AD Baseline, and AD One-Year networks respectively. Interestingly, these results are inconsistent with what has been seen in the literature, which has often found a higher participation coefficient in HC subjects than in AD patients^45,46^. This discrepancy may be explained due to using different number of nodes and a different parcellation scheme used in our analysis. Specifically, unlike many studies that examine participation coefficient under data-defined modules, our study used the pre-defined RSN modules of^30^. Our study also uses a larger number of brain regions (264 nodes under the Power atlas) compared to than many other studies. Although contrary to the findings in existing literature, a smaller participation coefficient under our method is actually more consistent with increased small-worldedness properties under the HC network and greater randomness under the AD network. In particular, a higher participation coefficient may be explained by a greater number of connections between RSNs compared to within RSNs under the AD network that is indicative of a greater randomness in the network.

### Node-specific disruptions

**Figure 7:**
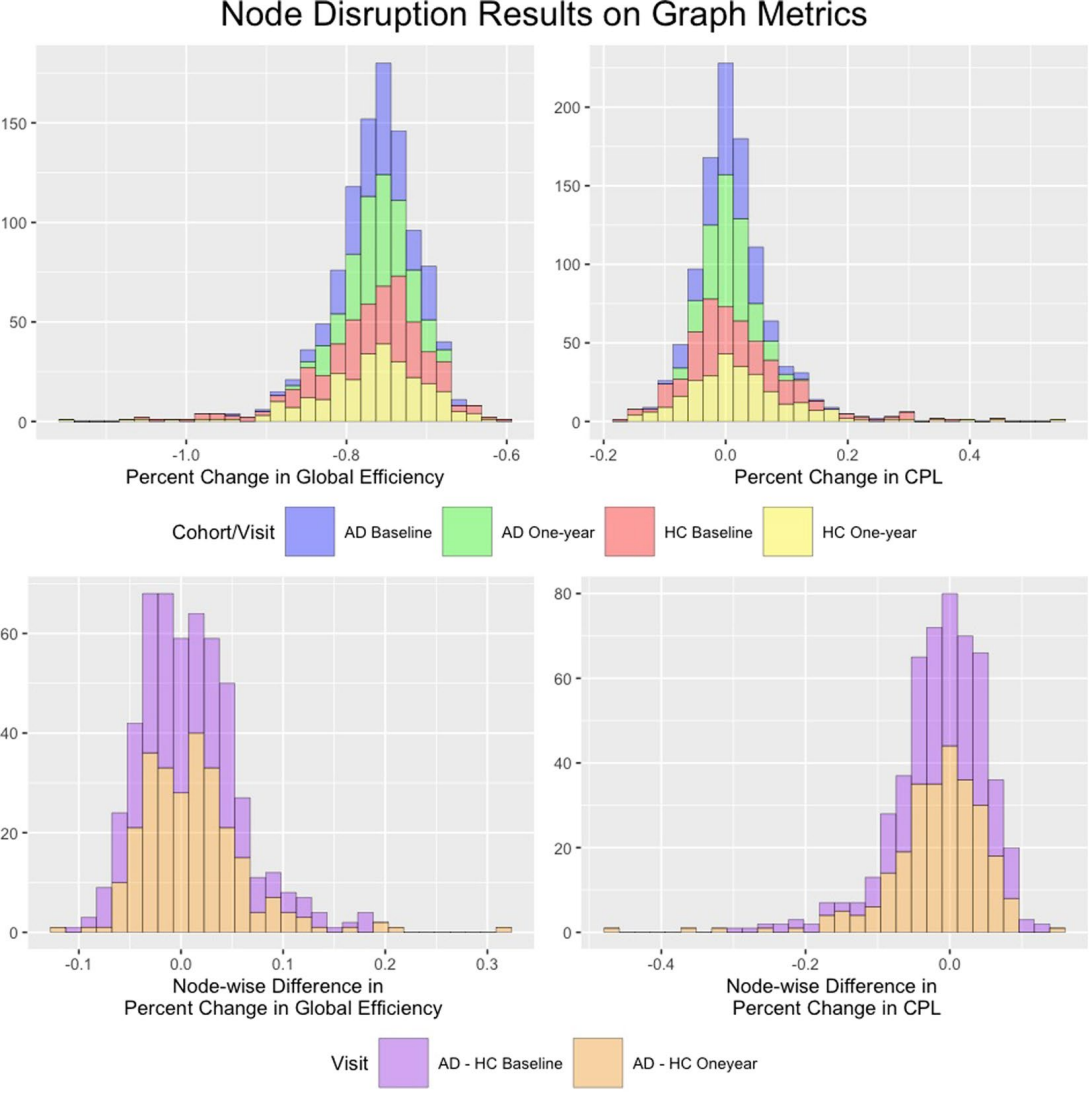
Global Efficiency and CPL results for the node disruption analysis. The top row plots histograms of the percent change in each metric after removing each node. The bottom row displays histograms of the node-specific difference between the percent change the the AD cohort and the percent change in the HC cohort across all nodes. Because all percent changes in GE were negative upon removal of a node, a positive value of this difference indicates that global efficiency was more affected in HC when that particular node was disconnected. Similarly, a negative value indicates that GE was more disrupted in AD network compared to the HC network when this node was disconnected. negative values for the difference in percent change in CPL between the AD and HC network upon disconnection of a node indicates that CPL increased more in the HC network.

The results of the node disruptions are presented in Fig. 7, which illustrates the histogram of the percent change in the metrics when one node is removed (top row), and the histogram of the percent change at the node level between the cohorts at each visit (bottom row). On the whole brain level, individual node removals had larger reductions in GE and CPL for the HC network (as evidenced by longer tails) which implies greater disruptions. This is indicative of the importance of hub nodes in the HC network that results in greater clustering tendency and lesser randomness compared to the AD network. To compare the node-level disruptions across cohorts we also plotted the difference in the percent changes in GE and CPL between the AD and the HC networks (bottom row in Fig. 7). Examination of these node-wise difference show that there are several nodes with relatively large differences between AD and HC at both baseline and one-year follow-up, and that attacks on these nodes disrupt the GE and CPL in the HC network more than for the AD network, as evidenced by longer tails in Fig. 7.

**Figure 8:**
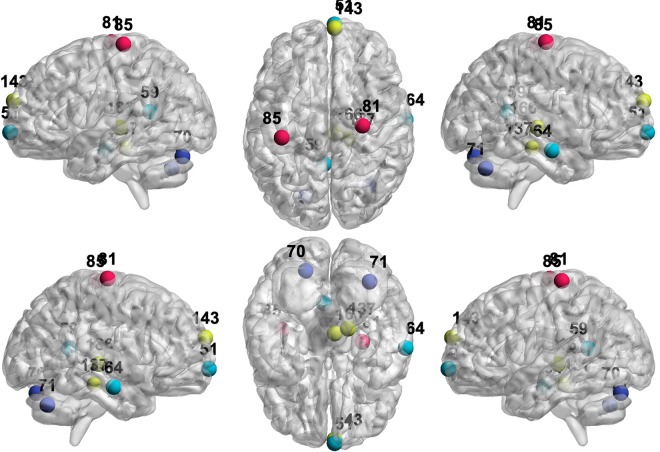
Locations of the nodes with the top 10 largest percent change in GE upon removal in HC and AD under BJNL. The nodes are colored by resting state network. Most of these nodes also exhibited strong differences in the magnitude of the percent change between AD and HC at baseline and one-year. See Table 2 for the corresponding values.

**Figure 9:**
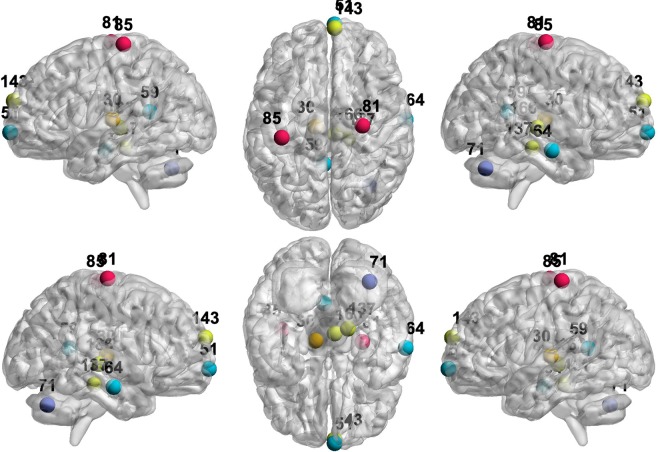
Locations of the nodes with the top 10 largest percent change in CPL upon removal in HC and AD under BJNL. The nodes are colored by resting state network. Most of these nodes also exhibited strong differences in the magnitude of the percent change between AD and HC at baseline and one-year. See Table 3 for the corresponding values.

The 10 nodes with the largest disruption effects for the HC cohort is displayed in Tables 2 and 3. Figures 8 and 9 display the corresponding locations in the brain. Interestingly, all of these nodes responsible for the greatest disruptions in GE are also hub nodes in Table 5. Similarly, all nodes resulting in the greatest disruption in CPL upon disconnection are also hub nodes in Table 5 and nine of these hub nodes are also important disruptors of GE in Table 2. These results support our hypothesis that the organization of the HC network is primarily dependent on a set of hub nodes, which when removed cause the largest disruptions in information transmission, and is supportive of the small-worldedness properties of the HC network. We note that the nodes with the highest disruptive power in terms of GE and CPL are all contained in the DMN, EC, SM, and Occ Pole RSNs, which also contain the largest number of hub nodes in the HC network in Table 5.

### Comparison with graphical lasso analysis

**Figure 10:**
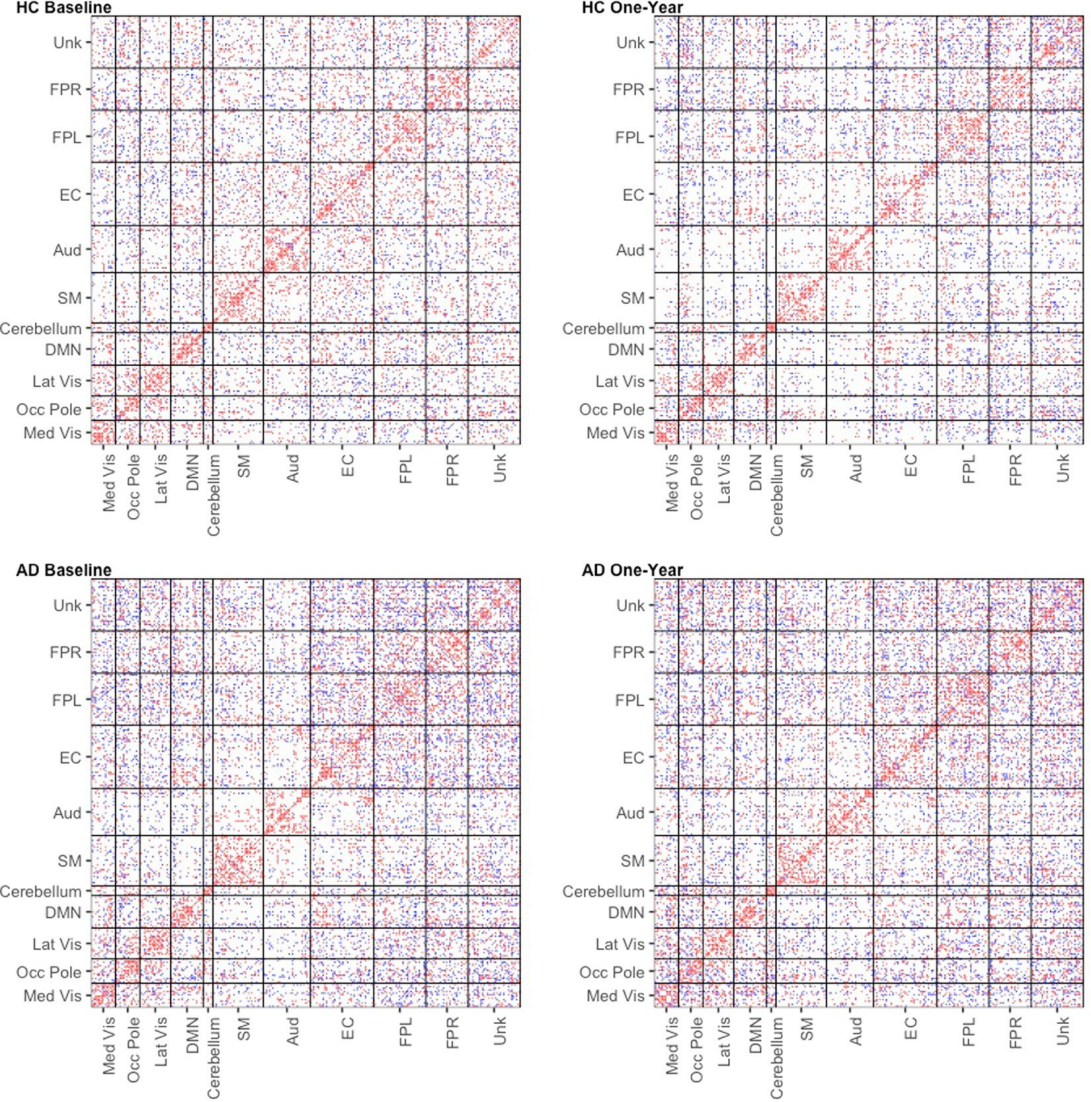
Estimated adjacency matrices for HC and AD at baseline and one-year follow-up using the graphical lasso. The edges are colored by the sign of the partial correlation (blue = negative, red = positive).

As an illustrative comparison, we conducted a similar analysis using the graphical lasso^47^, which estimates each cohort- and visit-specific network separately. We selected the tuning parameters of the graphical lasso to achieve network densities similar to the BJNL results, for a meaningful comparison. Figure 10 displays the estimated adjacency matrices from this approach. It is apparent that the graphical lasso estimates networks with a very different structure, and both the AD and HC networks seem to have more uniformly distributed connections that is indicative of a random network. Unlike the networks identified using BJNL which primarily had connections within RSNs, the graphical lasso produced network estimates with a significantly larger number of the edges are between resting state networks. For the HC network, only 17.3% and 18.9% of the edges belonged to within RSN connections, whereas for the AD network, the corresponding numbers were 15.8% and 15.9%. It is evident that there is almost a 50 percent reduction in the number of within RSN connections for the HC network under Glasso compared to BJNL, and similar large reductions are also observed in the AD network. This is potentially indicative of a more diffuse information transmission and decreased efficiency in network organization.

A large number of between RSN connections affect the ability to determine differences in the small-world network organization of the brain. Under the graphical lasso, point estimates of the small-worldedness in HC were 1.29 and 1.49 at baseline and one-year respectively, whereas those under the AD network were 1.30 and 1.22 at baseline and one-year respectively. Clearly, the separation between the small-worldedness values between the AD and HC networks is not nearly as distinct as it was for BJNL. The above findings point to a potentially reduced power under the graphical lasso to detect differences in small-worldedness between the AD and HC networks.

We also note that unlike BJNL, the HC networks at baseline and one-year under the graphical lasso illustrate weak reproducibility in terms of several network metrics. Under the graphical lasso analysis, the ICC values for betweenness, participation coefficient, nodal degree, local efficiency, and clustering coefficient for the HC network are 0.74, 0.63, 0.84, 0.11, and 0.39 respectively. While the ICC values for the first three metrics are reasonable, those for local efficiency and clustering coefficients are very weak, pointing to the difficulties of obtaining reproducible networks under the graphical lasso. This is potentially caused due to the more random nature of the estimated networks under the graphical lasso, and the inability of the approach to pool information across visits.

Finally, Tables 7 and 8 display the global efficiency and characteristic path length for a node disruption analysis under the graphical lasso, which is identical to the analysis conducted under BJNL. Figures S1 and S2 in the Supplementary Materials display the corresponding locations in the brain. Unlike the BJNL results in Table 2, the disruption in GE upon disconnection of a node is much more identical between the AD and HC network at baseline, implying greater overlap and smaller differences. Additionally, the CPL results in Table 8 show more nuanced disruptions under the graphical lasso. In particular, the sign of the changes in both GE and CPL can be both positive and negative, implying inconsistencies in the direction of change. Moreover, the maximum disruption potential for CPL under the graphical lasso analysis often do not coincide with the hub-nodes. These results potentially imply less meaningful interpretations under the alternative graphical lasso analysis that fail to elucidate the small-worldedness properties and highlight the role of hub nodes in the HC network, and does a relatively poor job of finding differences between the HC and the AD networks.

**Table 7:** Top 10 largest percent change in global efficiency upon removal of a node in HC and AD using the graphical lasso.

Node	RSN	HC Baseline	HC One-year	AD Baseline	AD One-year	AD − HC Baseline	AD − HC One-year
207	FPR	−0.83	−0.93	−0.86	−0.82	−0.02	0.12
259	Unk	−0.79	−0.84	−0.92	−0.89	−0.13	−0.04
252	Unk	−0.79	−0.91	−0.86	−0.85	−0.07	0.06
149	EC	−0.8	−0.88	−0.89	−0.8	−0.09	0.09
211	FPR	−0.8	−0.89	−0.79	−0.77	0	0.12
51	DMN	−0.81	−0.82	−0.88	−0.81	−0.07	0.01
143	EC	−0.8	−0.88	−0.83	−0.79	−0.03	0.09
195	FPL	−0.84	−0.88	−0.88	−0.85	−0.04	0.03
208	FPR	−0.76	−0.88	−0.81	−0.82	−0.05	0.06
164	EC	−0.82	−0.84	−0.88	−0.79	−0.06	0.05

Positive (negative) values indicate that removal of this node increased (decreased) the global efficiency. The final two columns display the difference in percent change between AD and HC at that node at baseline and at one-year.

**Table 8:** Top 10 largest percent change in CPL upon removal of a node in HC and AD using the graphical lasso.

Node	RSN	HC Baseline	HC One-year	AD Baseline	AD One-year	AD − HC Baseline	AD − HC One-year
*128	Aud	−0.01	−0.2	−0.09	−0.07	−0.08	0.13
*107	Aud	−0.02	−0.18	−0.06	−0.08	−0.04	0.1
*114	Aud	0	−0.09	−0.17	−0.08	−0.17	0.01
*123	Aud	−0.01	−0.09	−0.17	−0.08	−0.16	0.01
207	FPR	0.06	0.17	0.07	0.04	0.01	−0.13
110	Aud	0	0.15	0.03	0	0.03	−0.14
252	Unk	0.02	0.12	0.07	0.07	0.05	−0.06
149	EC	0.03	0.12	0.09	0.03	0.06	−0.09
169	EC	0.02	0.12	0.03	0.03	0.01	−0.09
164	EC	0.04	0.07	0.12	0.02	0.08	−0.04

Positive (negative) values indicate that removal of this node increased (decreased) the CPL. The final two columns display the difference in percent change between AD and HC at that node at baseline and at one-year. A *before a node indicates that it was not a hub in any of the cohorts/visits, as identified by the graphical lasso.

## Discussion

There was a clear increase in the number of connections between RSNs in AD as compared to HC under BJNL. Interestingly, it appears that the majority of these connections were strongly negative in the AD network. The increase in between-RSN edges in the AD network is consistent with the hypothesis that the AD brain behaves more like a random network than a small-world network. This behavior of the AD brain networks has been hypothesized to be potentially due to disruption of hub nodes in the brain^41^. We discovered the higher nodal disruptions in GE and CPL under the HC network, compared to the AD network, and noted that the hub nodes were responsible for the greatest disruptions in GE and CPL. This is not surprising since the HC network has many more hub nodes and exhibits greater small-worldedness than the AD network that is much closer to a random network. Moreover, the decrease in the number of hub nodes and small-worldness under the AD network from baseline to one year, in contrast to a minimal change in the HC network with respect to these metrics, suggests progression of the AD disease within a span of one-year. In addition, the BJNL analysis discovered a much higher agreement for hub nodes across visits under the HC network (Fig. 1) and also indicated high ICC values for several network metrics, which suggests strong reproducibility for the HC network across visits. On the other hand, an alternate analysis that separatel analyzes data from each visit using graphial lasso discovered less meaningful differences between HC and AD cohorts and a weaker agreement for hub nodes in the HC cohort between two visits (Table 9).

One possible source of heterogeneity is the presence of amyloid, which has been associated with changes in functional connectivity. In our analysis, all of the AD patients were amyloid positive, while the HC cohort was split into 19 amyloid negative and 7 amyloid positive individuals. To examine whether amyloid status influences the HC network, we performed an additional subject-level analysis where we used the graphical lasso to estimate the brain network for each subject in the HC group at each visit separately. Then, we fit models using amyloid status to predict global efficiency, characteristic path length, and mean clustering coefficient at baseline and one-year follow up. Amyloid status was not a statistically significant predictor of any of these metrics after correcting for multiple testing. As a follow-up analysis, we used BJNL to jointly estimate the brain networks for both amyloid based subgroups at baseline and at one-year. We then examined the distribution of partial correlation differences between groups at each time point, corrected for multiple comparisons. We found that there were 10.8% and 11.5% edges with significantly different strengths between the two groups at baseline and one year respectively. The results of the two analyses suggest that there are some differences between the amyloid positive and negative groups in connectivity strengths, but these differences in edge strengths do not dramatically alter the overall patterns of information transmission in the brain as encapsulated via network metrics.

Although the main focus in this article is finding brain network-based differences between AD and HC cohorts, we also note that another topic of interest in literature is the brain networks of MCI subjects. We performed a supplementary analysis of early and late MCI amyloid positive individuals (14 EMCI and 14 LMCI) that is similar to the analysis that was performed for the HC and AD patients. We saw a general increasing trend in the number of edges as impairment progressed from healthy to Alzheimer’s. We also saw some network metric differences between the MCI group and the AD group, and these MCI network metrics often seemed to lie in between the HC and the AD network metrics indicating some progression of the disorder in the MCI cohort. The details for this analysis can be found in the Supplementary Materials. Another feature of the MCI network is the pattern of reduced between-network anticorrelation compared to healthy elders^48^, particularly between seed voxels present in the default mode network (DMN) and the dorsal attention network (DAN). We performed additional analysis to examine whether the anticorrelation (measured as the strength of negative connections) between the DMN (58 nodes) and the DAN (11 nodes) under the Power atlas, was reduced in the EMCI and LMCI groups compared to the HC cohort at baseline and one-year follow up. We did not find significantly reduced anticorrelation in any of the cohorts relative to the other cohorts. However, when we expanded the analysis to include both amyloid positive and negative MCI individuals (24 EMCI and 23 LMCI), our results revealed a significantly reduced anticorrelation in the EMCI network compared to the HC network. Hence our region level analysis seems at least partially support existing evidence of reduced anticorrelation in MCI individuals Table 9.

**Table 9:** Hubs in each cohort and visit identified using a normalized betweenness score 1.5 standard deviations above the mean based on the graphical lasso.

Cohort/Visit	Node Index of Hub Nodes
HC - Baseline	27, 51, 59, 69, 112, 132, 137, 165, 166, 168, 175, 195, 207, 214
HC - One-Year	9, 17, 18, 21, 30, 50, 59, 64, 81, 110, 132, 137, 143, 149, 164, 166, 169, 177, 180, 182, 183, 184, 195, 199, 207, 208, 211, 214, 218, 235, 242, 244, 249, 252, 259
AD - Baseline	9, 20, 51, 137, 149, 164, 165, 175, 188, 195, 199, 207, 214, 221, 228, 235, 246, 252, 259
AD - One-Year	9, 124, 132, 195, 245, 246, 252, 257, 259

Finally, we note that there is a growing consensus among neuroradiologists that brain hypoperfusion is likely involved in the pathogenesis of AD and that disturbed cerebral blood flow (CBF) can serve as a promising biomarker for predicting conversion of mild cognitive impairment to AD^49,50^. In future work, it would be interesting to examine if these cerebral haemodynamic changes are correlated with loss of small-worldedness properties in AD individuals, compared to the HC cohort. Such an effort would be instrumental in directly relating cerebral brain health with changes in the brain network topology, which could be of potential translational and clinical significance. We plan to investigate these questions in future work.

## Supplementary information

Supplementary Materials

## Data Availability

Data used in our analysis were downloaded from the ADNI website (http://www.adni.loni.usc.edu) and is publicly available. The code used to produce the results will be made available by the authors on reasonable request.
