# Supplementary material for: A Novel Joint Brain Network Analysis Using Longitudinal Alzheimer’s Disease Data
Source: Sci Rep. 2019 Dec 20;9:19589. doi: 10.1038/s41598-019-55818-z (PMC6925181; doi:10.1038/s41598-019-55818-z)
Supplement: Supplementary file 1 — Supplementary Materials [file 41598_2019_55818_MOESM1_ESM.pdf]

Supplementary Materials for A Novel Joint Brain Network Analysis Using Longitudinal Alzheimer's Disease Data

Suprateek Kundu, Joshua Lukemire, Yikai Wang, Ying Guo, and for the Alzheimer's Disease Neuroimaging Initiative

Department of Biostatistics and Bioinformatics, Emory University, Atlanta, Ga 30322, USA.

Rs-fMRI data acquisition and pre-processing

A T1-weighted high- resolution anatomical image (MPRAGE) and a series of resting state functional images were acquired with a 3.0 Tesla MRI scanner (Philips Systems) during longitudinal visits. The rs-fMRI scans were acquired with 140 volumes, TR/TE = 3000/30 ms, flip angle of 80 and effective voxel resolution of $3.3 \times 3.3 \times 3.3$ mm. More details can be found at the ADNI website (*http://www.adni.loni.usc.edu*). Quality control was performed on the fMRI images both by following the Mayo clinic quality control documentation (version 02-02-2015) and by visual examination. After the quality control, 44 subjects were included for the analysis, with 27 subjects in the healthy group and 17 subjects in the AD group. The HC group had 55.6% females with a mean age of 74.02 at baseline and that of 75.08 at one year follow-up. The AD group has 58.8% females with the mean age at baseline being 73.91 and at one year follow-up being 74.94. The median ages in the healthy and Alzheimer's disease groups at baseline were 73.80 and 74.50 respectively. There are no significant differences in terms of age (t-test, $t = 0.05$, $p = 0.96$) and sex (Fisher's exact test, $p = 1.00$) between the two cohorts. The education status information is measured in number of years in the ADNI dataset. The mean (sd) of years of education was 16.26 (2.12) and 14.88 (2.62) in the HC and AD groups, respectively. We see no significant difference with respect to this variable between the AD and HC cohorts (t-test, $t = 1.82$, $p = 0.08$). We observe that 37% and 88% of the subjects have at least one copy of APOE4 in the HC and AD cohorts, respectively. A Fisher's exact test revealed that the proportion of individuals with the APOE4 present is significantly higher in the AD cohort compared to the HC cohort ($p = 0.001$). All AD subjects included in the analysis were amyloid positive, and 7 of the 27 HC subjects were also amyloid positive. We identified amyloid positivity using the technique described in [1]. Specifically, we calculated florbetapir SUVRs by averaging across the 4 cortical regions and dividing this cortical summary ROI by a composite reference region. We follow the suggestion from ADNI to use a cutoff at 0.79 for Amyloid positivity.

The data was pre-processed using standard pipelines as follows. Skull stripping was conducted on the T1 images to remove extra-cranial material. The first 4 volumes of the fMRI were removed to stabilize the signal, leaving 136 volumes for subsequent prepossessing. We registered each subject's anatomical image to the 8th volume of the slice-time-corrected functional image, and then the subjects' images were normalized to MNI standard brain space. Spatial smoothing with a 6mm FWHM Gaussian kernel and motion corrections were applied to the function images. A validated confound regression approach [2, 3] was performed on each subject's time series data to remove the potential confounding factors including motion parameters, global effects, white matter (WM) and cerebrospinal fluid (CSF) signals. Furthermore, motion-related spike regressors were included to bound the observed displacement and the functional time series data were band- pass filtered to retain frequencies between 0.01 and 0.1 Hz which is the relevant range for rsfMRI. For fitting the BJNL approach, the data was prewhitened using an autoregressive model.

Network Metrics

A description of the network metrics used for the analysis is provided below.

Global Efficiency A measure of information transmission across the entire brain calculated by averaging the inverse shortest path lengths across all brain nodes. Higher values of global efficiency indicate more efficient information transmission.

Local Efficiency A node-specific version of global efficiency. We examined the average local efficiency over all nodes in the brain.

Characteristic Path Length (CPL) A measure similar to global efficiency, the CPL is calculated by averaging the shortest path length across all pairs of nodes in the brain. Smaller values of CPL indicate that on average a smaller number of intermediate steps are required for a pair of nodes to communicate.

Mean Clustering Coefficient (MCC) A measure of the interconnectedness of the brain network calculated by counting how many of a brain node's neighbors are also neighbors of each other, averaged over all nodes.

Small-Worldedness A measure of whether or not the brain exhibits small world properties calculated by examining the ratio of normalized mean clustering coefficient to normalized characteristic path length, $SW = \frac{MCC/MCC_0}{CPL/CPL_0}$. Here MCC_0, CPL_0, refer to the metrics corresponding to a baseline distribution for each cohort and visit by generating 1000 surrogate random networks with the same connection density as the estimated graph for the cohort/visit and then calculating the average CPL and MCC for these surrogate graphs. The ratio of each metric to the average over the surrogate graphs is then taken as the normalized metric. A ratio $SW > 1$ indicates that the estimated brain network exhibits more small-worldedness than a random network.

Betweenness A node-specific measure of importance, betweenness examines how frequently a node is a part of the shortest path between two *other* nodes. High values of betweenness suggest that a node may be a hub node, as a large number of the optimal paths of information transmission pass through it. We calculated a normalized betweeenness metric by dividing by the average betwenness within each cohort/visit [4].

Participation Coefficient Another node-specific measure of importance, participation examines the proportion of a node's connections that are to RSNs other than the one the node is a member of. Large values of the participation coefficient indicate that a significant amount of the node's communication is with other RSNs of the brain. For this metric, only the 232 nodes belonging to a known RSN were used.

Description of Bayesian Joint Network Learning

Suppose $\mathbf{y}_{g,it}$ denotes the p-dimensional vector of prewhitened fMRI measurements over p brain regions ($p = 264$ for the Power atlas) for the t-th brain volume at baseline ($g = 0$) and one year follow-up ($g = 1$) for the i-th subject ($i = 1, \ldots, n$). Our analysis jointly estimates the networks at baseline and one-year follow-up for AD subjects and then performs the analysis separately for HC subjects. We model

$$\mathbf{y}_{g,it} \sim N_p(\mathbf{0}, \Omega_g^{-1}),\ \Omega_g^{-1} \in M_p^+,\ i = 1, \ldots, n, t = 1, \ldots, T, \tag{1}$$

where N_p denotes a p-dimensional Gaussian distribution, M_p^+ denotes the space of all symmetric and positive definite matrices, and Ω_g captures the sparse inverse covariance or precision matrix encoding the network at longitudinal visit $g = 0, 1$. The BJNL approach specifies spike and slab graphical lasso priors on the inverse covariance off-diagonal elements, and Exponential type priors on the diagonal elements of the precision matrix. The prior specification is designed to ensure that the precision matrices are drawn from M_p^+, the space of all positive-definite precision matrices. The spike and slab graphical lasso priors on the precision off-diagonals ensure that these elements are assigned small absolute values close to zero corresponding to unimportant edges (under a Laplace type specification) with some probability π, and large absolute values corresponding to significant edges in the network with probability $1 - \pi$. These edge inclusion probabilities are edge-specific and network-specific, and are constructed as flexible functions of a shared component that is common across networks and differential components that are network-specific. Each edge inclusion probability is jointly estimated by pooling information across the baseline and one-year follow-up, which results in the joint estimation of the longitudinal networks. Once these inclusion probabilities are estimated, the elements of the precision matrices can be sampled using closed form posterior distributions. The edge-inclusion probabilities are thresholded in a systematic manner in order to infer important connections, which is designed to control the false discovery rate and yield meaningful estimates for the network.

Node disruption diagrams under graphical lasso

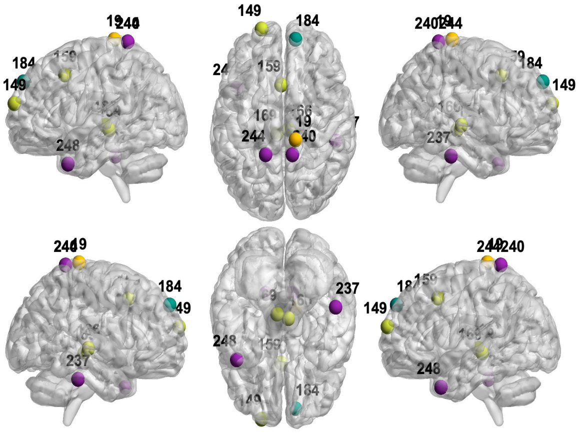

Figure S1. Locations of the nodes with the top 10 largest percent change in GE upon removal in HC and AD identified using the graphical lasso. The nodes are colored by resting state network. These nodes exhibited very small changes in the magnitude of the percent change between AD and HC at baseline and one-year and in several cases there were no differences identified at all.

Table S1. Number of edges in the brain network for each cohort at baseline and one-year follow-up.

	Baseline	One-Year
HC	6444	6029
EMCI	7060	6934
LMCI	6443	7048
AD	8049	8401

MCI Analysis

We analyzed the early MCI (EMCI) and late MCI (LMCI) patients using the same procedure as for the HC and AD patients. Only MCI patients who were amyloid positive were included in the analysis. There were 14 EMCI (9 males, average age 71.0, average education = 15.6 years) and 14 LMCI subjects (7 males, average age =72.1, average education = 16.7 years). We present some select results of the BJNL analysis below. Table S1 displays the number of edges in each group at baseline and one-year follow up. Figure S3 displays the corresponding adjacency matrices. Graph metric densities for each group and time point are presented in Figure S4. We see that, in general, the MCI patients are more similar to the HC subjects than to the AD patients.

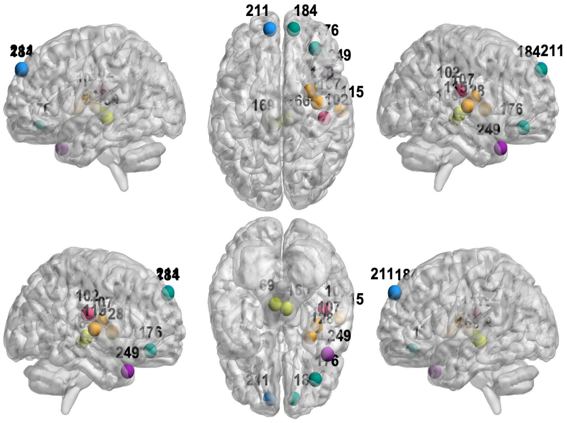

Figure S2. Locations of the nodes with the top 10 largest percent change in CPL upon removal in HC and AD identified using the graphical lasso. The nodes are colored by resting state network. These nodes exhibited very small changes in the magnitude of the percent change between AD and HC at baseline and one-year.

References

1. Landau, S. M. et al. Measurement of longitudinal β-amyloid change with 18f-florbetapir pet and standardized uptake value ratios. J. Nucl. Medicine **56**, 567–574 (2015).

2. Satterthwaite, T. D. et al. An improved framework for confound regression and filtering for control of motion artifact in the preprocessing of resting-state functional connectivity data. Neuroimage **64**, 240–256 (2013).

3. Satterthwaite, T. D. et al. Heterogeneous impact of motion on fundamental patterns of developmental changes in functional connectivity during youth. Neuroimage **83**, 45–57 (2013).

4. He, Y., Chen, Z. & Evans, A. Structural insights into aberrant topological patterns of large-scale cortical networks in alzheimer's disease. J. Neurosci. **28**, 4756–4766 (2008).

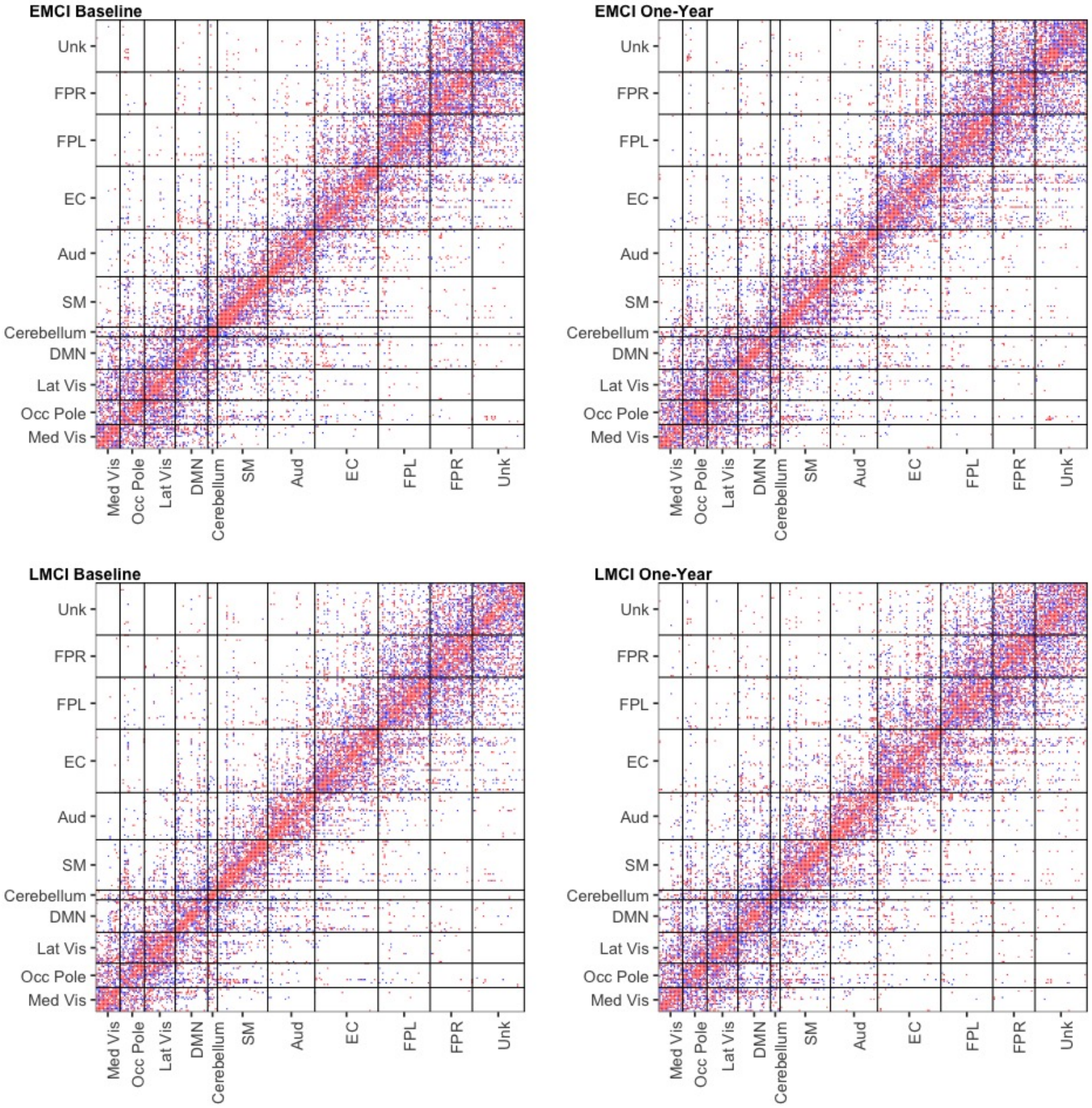

Figure S3. Estimated adjacency matrices for EMCI and LMCI at baseline and one-year follow-up using only amyloid positive MCI patients. The edges are colored by the sign of the partial correlation (blue = negative, red = positive). There are 7060 and 6934 estimated edges in EMCI at baseline and one-year follow-up respectively. There are 6443 and 7048 estimated edges in LMCI at baseline and one-year follow-up respectively. The RSNs are abbreviated follows: medial visual network (Med vis), occipital pole visual network (Occ pole), lateral visual network (Lat vis), default mode network (DMN), cerebellum (Cerebellum), sensorimotor (SM), auditory (Aud), executive control (EC), right frontoparietal (FPR), left frontoparietal (FPL), and unknown (Unk)

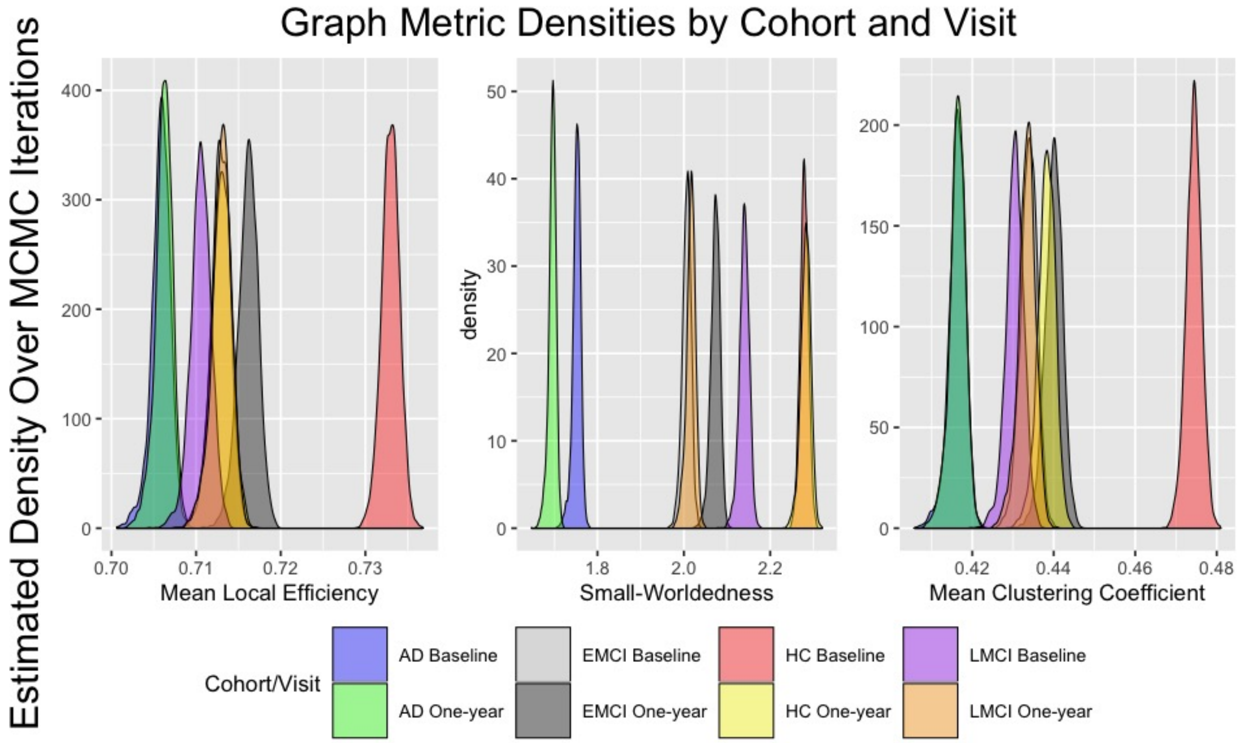

Figure S4. Graph metrics in the healthy controls (HC), early MCI (EMCI), late MCI (LMCI), and Alzheimer's disease patients (AD) at baseline and one-year follow up.
